# Modulating the gut ecosystem dietary, probiotic, and novel interventions for bone health in postmenopausal women

**DOI:** 10.3389/fimmu.2026.1814866

**Published:** 2026-07-03

**Authors:** Ying Lin, Jiayong Zheng, Dongquan Hu, Yaozhe Ying, Yuan Zhu, Zheyan Chen

**Affiliations:** 1 Department of Maternal and Child Health, Wenzhou Maternal and Child Health Care Hospital, Third Clinical Institute Affiliated to Wenzhou Medical University, The Third Affiliated Hospital of Shanghai University, Wenzhou People’s Hospital, Wenzhou, China; 2 Department of Medical Genetics, The Third Clinical Institute Affiliated to Wenzhou Medical University/The Third Affiliated Hospital of Shanghai University/Wenzhou People’s Hospital/Wenzhou Maternal and Child Health Care Hospital, Wenzhou, China; 3 Department of Clinical Nutrition Center, Wenzhou Third Clinical Institute Affiliated to Wenzhou Medical University, Wenzhou People’s Hospital, Wenzhou, China; 4 Department of Plastic and Reconstructive Surgery, Wenzhou Third Clinical Institute Affiliated to Wenzhou Medical University, The Third Affiliated Hospital of Shanghai University, Wenzhou People’s Hospital, The Wenzhou People’s Hospital Affiliated of Hangzhou Medical College, Wenzhou, China

**Keywords:** bone metabolism, brain-gut-bone axis, gut microbiota, immune regulation, postmenopausal osteoporosis, probiotics, short-chain fatty acids

## Abstract

Postmenopausal osteoporosis (PMO) is a metabolic bone disorder caused by estrogen deficiency, posing significant risks to the skeletal health and quality of life of middle-aged and elderly women. In recent years, the gut microbiota (GM) has emerged as a novel regulatory target in bone metabolism, attracting increasing research interest. Probiotics may modulate bone metabolism by directly introducing beneficial microorganisms (e.g., Lactobacillus, Bifidobacterium) to improve gut microbiota composition. The gut microbiota may influence the onset and progression of osteoporosis by modulating immune-inflammatory responses, endocrine regulation, nutrient absorption, and the production of metabolic byproducts. This review systematically summarizes the mechanisms by which gut microbiota affects postmenopausal osteoporosis, including the neuroendocrine brain-gut-bone axis, immune regulation, metabolic products such as short-chain fatty acids, intestinal barrier function, and their correlations with bone mineral density. Integrating the latest clinical and animal model studies, we further explore gut microbiota-based intervention strategies, such as probiotics, prebiotics, fecal microbiota transplantation, and dietary modulation. These insights may provide a theoretical foundation and practical guidance for the prevention and treatment of postmenopausal osteoporosis, highlighting the promising role of gut microbiota-targeted therapies in improving bone health in postmenopausal women.

## 1 Introduction

Postmenopausal osteoporosis (PMO) is a significant public health concern, particularly affecting women after menopause due to the decline in estrogen levels, which may leads to increased bone resorption and decreased bone formation. This condition results in a reduction of bone mass and microarchitectural integrity, heightening the risk of fractures. With the aging global population, the prevalence of PMO is expected to rise, necessitating effective preventive and therapeutic strategies. Traditional treatments, including hormone replacement therapy and bisphosphonates, have been widely used; however, they are often associated with significant adverse effects. For instance, hormone replacement therapy may increase the risk of breast cancer and venous thromboembolism, while bisphosphonates can lead to gastrointestinal disturbances and, in rare cases, atypical femoral fractures (1). Furthermore, the administration routes of these treatments (e.g., frequent oral dosing or periodic intravenous infusions) often result in poor patient compliance. Consequently, there is an urgent need for alternative approaches. Strategies based on gut microbiota offer a promising and potentially safer avenue for intervention, as they may modulate bone health through natural metabolic pathways with fewer severe side effects and greater patient convenience, in contrast to the aforementioned pharmacological treatments. Recent research has begun to explore the intricate relationship between gut microbiota and bone health, suggesting that the gut microbiome may play a pivotal role in the pathophysiology of PMO, thereby offering new avenues for intervention. This suggestion is primarily grounded in the “gut-bone axis” hypothesis, which posits that the gut microbiota can systemically influence bone metabolism through several key mechanisms, including immune modulation, the production of metabolic products (such as short-chain fatty acids), and endocrine pathways (2, 3).

The gut microbiota, a complex community of microorganisms residing in the gastrointestinal tract, has been shown to influence various aspects of human health, including metabolic processes, immune functions, and even bone metabolism. Dysbiosis, or an imbalance in gut microbiota composition, has been implicated in several metabolic bone diseases, including osteoporosis (3). Recent studies have identified specific microbial taxa and their metabolic products, such as short-chain fatty acids (SCFAs), as key modulators of bone health (4, 5). SCFAs, produced through the fermentation of dietary fibers by gut bacteria, have anti-inflammatory properties and can enhance the absorption of calcium, thereby potentially influencing bone density and strength (6–8).Furthermore, the gut microbiota interacts with the immune system, impacting the inflammatory processes that are crucial in the development of PMO.

The connection between gut microbiota and bone health can be conceptualized through the gut-bone axis, which encompasses various mechanisms, including the modulation of immune responses and the production of metabolites that affect bone remodeling. For instance, SCFAs such as butyrate have been shown to promote osteoblast differentiation and inhibit osteoclast activity, thus favorably influencing bone formation and resorption (9).Additionally, gut-derived metabolites can impact the systemic inflammatory environment, which is particularly relevant in the context of PMO, where chronic inflammation is often observed. This highlights the potential of targeting gut microbiota as a therapeutic strategy for managing PMO (10, 11).

Emerging evidence suggests that dietary interventions, such as the incorporation of prebiotics and probiotics, can positively influence gut microbiota composition and function, leading to improved bone health outcomes. Prebiotics, which serve as food for beneficial gut bacteria, can enhance microbial diversity and promote the production of SCFAs (12).Probiotics, on the other hand, constitute beneficial microorganisms including not only bacteria but also beneficial yeasts such as Saccharomyces boulardii. Clinical studies have begun to explore the effects of these interventions on bone mineral density and fracture risk in postmenopausal women, yielding promising results that warrant further investigation.

This review aims to provide a comprehensive overview of the current understanding of the relationship between gut microbiota and postmenopausal osteoporosis, exploring the underlying mechanisms that connect these two entities drawing on evidence from both preclinical and clinical studies. We will discuss the role of specific microbial taxa, the impact of dietary interventions, and the potential of gut microbiota modulation as a therapeutic strategy for improving bone health in postmenopausal women. By elucidating these connections, we hope to contribute to the development of innovative and effective treatment approaches for PMO, ultimately enhancing the quality of life for affected individuals.

## 2 The association mechanisms between postmenopausal osteoporosis and gut microbiota

### 2.1 Neuropeptide Y-mediated brain-gut-bone axis mechanism

Postmenopausal osteoporosis (PMO) is characterized not only by bone loss but also by significant neuroendocrine alterations within the hypothalamus, which may play a crucial role in modulating bone metabolism through the brain-gut-bone axis. Among the neuroendocrine factors, Neuropeptide Y (NPY) has been suggested as a pivotal mediator linking hypothalamic changes to gut microbiota (GM) dynamics and subsequent bone health. In ovariectomized (OVX) rat models that simulate the postmenopausal state of estrogen deficiency, the lack of estrogen leads to NPY overexpression in the hypothalamus, thereby activating the brain-gut-bone axis.Recent research has elucidated that NPY overexpression in ovariectomized (OVX) rat models, which simulate postmenopausal estrogen deficiency, leads to a marked reduction in bone formation and deterioration of bone microarchitecture. This is accompanied by an upregulation of pyroptosis-related proteins in subchondral cancellous bone, indicating that NPY can not only impairs osteoblastic activity but also induces inflammatory cell death pathways in bone tissue (13).

Concomitantly, NPY overexpression exacerbates colonic inflammation and compromises intestinal barrier integrity, by downregulating the expression of tight junction proteins (such as claudin, occludin, and zonula occludens−1) and mucin, and increasing the infiltration of colonic inflammatory cells, resulting in increased intestinal permeability. This disruption facilitates the translocation of bacterial endotoxins such as lipopolysaccharide (LPS) into systemic circulation, which further promotes inflammatory responses detrimental to bone remodeling. Elevated serum LPS levels have been shown to induce pyroptosis in osteoblasts, reducing their viability and differentiation potential, thereby impairing bone formation and accelerating osteoporosis progression (13). These findings underscore the integral role of gut barrier function and microbial metabolites in the pathophysiology of PMO mediated by neuroendocrine factors.

Importantly, the administration of Y1 receptor antagonists (Y1Ra) effectively reverses the pathological changes induced by NPY overexpression. Y1Ra treatment restores bone formation rates, ameliorates bone microstructure damage, and suppresses pyroptosis in bone cells. Additionally, Y1Ra mitigates colonic inflammation, enhances intestinal barrier integrity, and reduces serum LPS concentrations, demonstrating a protective effect on gut permeability and systemic endotoxemia. This receptor blockade also modulates the diversity and composition of gut microbiota, shifting the microbial community toward a more balanced state, which correlates with improved bone health outcomes (13).

Fecal microbiota transplantation experiments further validate the causal relationship between NPY-mediated alterations in gut microbiota and bone deterioration, highlighting the brain-gut-bone axis as a critical pathway in PMO pathogenesis. These insights reveal that targeting the NPY-Y1 receptor signaling axis within this neuroendocrine-gut microbial network may represent a novel therapeutic strategy for managing postmenopausal osteoporosis. By modulating both central neuropeptide signaling and peripheral gut microbiota composition, it is possible to attenuate bone loss and improve skeletal integrity in affected individuals (13, 14). Thus, the NPY-mediated brain-gut-bone axis provides a promising new avenue for research and clinical intervention in PMO.

### 2.2 Estrogen deficiency and changes in gut microbial diversity

Estrogen deficiency following menopause is a critical factor that profoundly influences the gut microbial ecosystem, leading to significant alterations in microbial diversity, richness, and gut barrier integrity, which collectively contribute to the pathogenesis of postmenopausal osteoporosis (PMO). Studies utilizing ovariectomized (OVX) animal models, which simulate estrogen deficiency, have consistently demonstrated a marked reduction in alpha diversity of gut microbiota compared to controls, indicating a loss in microbial richness and evenness (15).This diminished diversity is accompanied by compositional shifts characterized by an increase in potentially pathogenic bacteria such as Helicobacter rodentium and a decrease in beneficial taxa like Lactobacillus and Prevotella species (15, 16). Estrogen deficiency directly impairs the expression of tight junction proteins (claudin, occludin, and zonula occludens−1) and mucin, thereby disrupting intestinal epithelial structure and elevating gut permeability. These changes compromise the intestinal barrier function, as evidenced by decreased expression of tight junction proteins (e.g., claudin, occludin, and zonula occludens-1) and mucin, which normally maintain gut epithelial integrity (17).The increased gut permeability facilitates translocation of bacterial endotoxins such as lipopolysaccharide (LPS) into systemic circulation, thereby promoting a state of low-grade chronic inflammation, or inflammaging, which is a recognized contributor to bone resorption and osteoporosis progression (18, 19).

Estrogen deficiency induces gut dysbiosis, creating a pro-inflammatory environment that exacerbates osteoclastogenesis through immune system activation. This dysbiotic state favors pro-inflammatory taxa and disrupts the Th17/Treg balance, promoting bone resorption (20–23). Conversely, estrogen replacement or hormone therapy in postmenopausal women can partially reverse these effects by restoring gut microbial diversity and enriching beneficial genera such as *Coprococcus* and *Eubacterium*, which are associated with lower bone turnover markers (20). The gut microbiota influences bone metabolism through multiple mechanisms, including the production of microbial metabolites like short-chain fatty acids (SCFAs) and the regulation of systemic inflammatory mediators (22, 23). Notably, specific bacterial species like *Prevotella histicola* have been identified to protect against estrogen deficiency-induced bone loss by enhancing gut barrier integrity and suppressing osteoclastogenic cytokines (24).

However, human studies reveal complexity and interindividual variability in the relationship between estrogen status and gut microbiota. Some investigations report minimal changes in gut microbial composition shortly after oophorectomy despite significant hormonal and bone density alterations, suggesting that factors such as body mass index and lifestyle may exert stronger influences on microbiota than estrogen alone (7).Nonetheless, longitudinal studies in postmenopausal women consistently demonstrate that estrogen deficiency correlates with decreased abundance of beneficial bacteria like Lactobacillus and increased microbial diversity associated with pathogenic taxa (25, 26).These microbial shifts are linked to altered metabolic pathways including tryptophan metabolism and bile acid signaling, which further impact bone health (27, 28).

In addition, estrogen deficiency reshapes microbial metabolic functions by reducing the abundance of SCFA−producing bacteria and disturbing bile acid deconjugation and metabolism. The reduced SCFA generation further weakens anti−inflammatory effects and calcium absorption efficiency, forming a vicious cycle among estrogen decline, gut dysbiosis, metabolic disorders, and bone loss.

In summary, estrogen deficiency precipitates a decline in gut microbial diversity and disrupts the composition of the gut microbiota, leading to compromised intestinal barrier function and systemic low-grade inflammation. These changes form a complex estrogen-gut microbiota-bone axis that modulates bone remodeling and contributes to osteoporosis development in postmenopausal women. Therapeutic interventions targeting this axis, including hormone replacement therapy, probiotics, prebiotics, and dietary modifications, hold promise for mitigating bone loss by restoring gut microbial balance and intestinal integrity (29–31). Further research is warranted to elucidate the precise microbial species and metabolic pathways involved, as well as to understand interindividual variability to optimize personalized treatment strategies.

### 2.3 Gut microbiota and immune-inflammatory regulation

Postmenopausal osteoporosis (PMO) is intricately linked to immune system imbalance, particularly involving the dysregulation of T helper 17 (Th17) cells and regulatory T (Treg) cells, which are pivotal in modulating inflammatory responses that affect bone metabolism. The gut microbiota plays a crucial role in maintaining the balance between Th17 and Treg cells, thereby influencing the expression of pro-inflammatory cytokines such as interleukin-17 (IL-17) and tumor necrosis factor-alpha (TNF-α). These cytokines are known to promote osteoclastogenesis and bone resorption, leading to bone loss in postmenopausal women. Dysbiosis of the gut microbiota can exacerbate this imbalance by enhancing the secretion of pro-inflammatory factors, which in turn activate osteoclasts and intensify bone degradation. For instance, an altered gut microbial composition can increase intestinal permeability and systemic inflammation, facilitating the translocation of microbial products that stimulate immune cells to produce IL-17 and TNF-α, thereby aggravating bone resorption (32).Conversely, beneficial microbes and probiotics have demonstrated the capacity to restore immune homeostasis by modulating the gut environment, suppressing excessive inflammatory responses, and promoting Treg cell differentiation. These immunomodulatory effects contribute to the inhibition of osteoclast activity and preservation of bone mass. Several studies have highlighted that specific probiotic strains and microbial metabolites, such as short-chain fatty acids (SCFAs), can enhance the intestinal barrier, reduce systemic inflammation, and improve bone density by regulating immune functions (33, 34).Moreover, traditional Chinese medicine formulations and bioactive compounds have been shown to exert protective effects against inflammation-induced bone loss by reshaping thegut microbiota and modulating immune responses, including downregulation of TNF-α and IL-17 (35, 36).The gut microbiota’s influence extends beyond local intestinal immunity, as it can affect systemic immune regulation through complex host-microbe interactions involving innate and adaptive immune cells, including macrophages, dendritic cells, and lymphocytes. This crosstalk is essential for maintaining bone homeostasis, especially in the context of estrogen deficiency after menopause. Therefore, targeting the gut microbiota to restore immune balance represents a promising therapeutic strategy for mitigating postmenopausal osteoporosis by attenuating inflammation-driven bone resorption and enhancing bone formation (37, 38).

### 2.4 The role of gut microbial metabolites in bone metabolism

Short-chain fatty acids (SCFAs), including acetate, propionate, and butyrate, are key metabolites produced by the fermentation activity of gut microbiota on dietary fibers. These metabolites have emerged as critical regulators of bone metabolism through multiple mechanisms (4, 39).

Acetate, the most abundant SCFA, mainly enhances intestinal calcium absorption by upregulating calcium transporter proteins (e.g., TRPV6, CaBP−9k) and activates GPR43 to suppress systemic inflammation, thereby indirectly reducing bone resorption (40, 41).

Propionate regulates bone homeostasis mainly by promoting Treg cell differentiation, inhibiting Th17 cell overactivation, and reducing IL−17 and TNF−α secretion, which further suppresses osteoclastogenesis (22, 42). Propionate also enhances gut barrier function and reduces LPS translocation to alleviate inflammation− driven bone loss (38, 43).

Butyrate directly acts on bone cells: it promotes osteoblast differentiation by upregulating Runx2 and Osterix, and inhibits osteoclast maturation by suppressing NFATc1 and cathepsin K even at low concentrations (40, 41). As a histone deacetylase inhibitor (HDACi), butyrate also epigenetically regulates bone metabolism–related genes and reinforces intestinal barrier function (40, 44).

SCFAs promote bone formation by enhancing osteoblast differentiation and activity while concurrently inhibiting osteoclastogenesis, thus maintaining bone homeostasis. Experimental evidence demonstrates that SCFAs suppress osteoclast differentiation by modulating precursor cell populations and altering gene expression profiles related to bone resorption. For instance, butyrate has been shown to inhibit osteoclast maturation even at low serum concentrations, suggesting a potent systemic effect of these microbial metabolites on skeletal remodeling (40).Furthermore, SCFAs act through G protein-coupled receptors (GPCRs) such as GPR43 on immune and bone cells, mediating anti-inflammatory effects and reinforcing gut barrier integrity, which indirectly supports bone health by reducing systemic inflammation (45).This dual action—direct modulation of bone cells and immune regulation—positions SCFAs as pivotal mediators in the gut-bone axis, offering promising therapeutic targets for osteoporosis, especially postmenopausal osteoporosis (PMO) where estrogen deficiency disrupts bone remodeling balance (41, 46, 47).

In patients with postmenopausal osteoporosis, serum and fecal levels of acetate, propionate, and butyrate are all significantly reduced, correlating positively with bone mineral density (BMD). Clinical and preclinical studies have consistently reported decreased bacterial diversity and altered gut microbial composition in PMO, with a concomitant decline in fecal and serum SCFA concentrations. These changes are associated with increased bone fragility and microstructural damage (48).For example, reductions in butyrate-producing bacteria such as Roseburia intestinalis have been linked to lower BMD and impaired bone microarchitecture. Metabolomic profiling in PMO patients reveals diminished SCFA abundance alongside alterations in other metabolites involved in amino acid and lipid metabolism, indicating a broad metabolic dysregulation connected to gut microbial shifts. The positive correlation between SCFA levels and BMD underscores the importance of these metabolites as biomarkers and potential modulators of bone health in estrogen-deficient states (49). Restoration of SCFA levels through dietary interventions, probiotics, or pharmacological agents has demonstrated improvements in bone parameters, reinforcing their therapeutic relevance (50–52).

Mechanistically, acetate, propionate, and butyrate synergistically regulate bone metabolism by modulating immune cell function and maintaining intestinal barrier integrity, thereby influencing systemic inflammation and bone remodeling. SCFAs enhance the differentiation and function of regulatory T cells (Tregs) while suppressing pro-inflammatory Th17 cells, which are implicated in osteoclast activation and bone resorption. This immunomodulatory effect helps restore the Th17/Treg balance disrupted in PMO, reducing inflammatory cytokines such as IL-17 and TNF-α that promote bone loss. Additionally, SCFAs strengthen the gut epithelial barrier by upregulating tight junction proteins, preventing translocation of microbial products that can trigger systemic inflammation detrimental to bone. Through activation of GPCRs like GPR43 and GPR41 on immune and bone cells, SCFAs initiate intracellular signaling cascades that inhibit osteoclastogenesis and promote osteoblast activity. These pathways also intersect with endocrine signals, including estrogen and vitamin D metabolism, integrating microbial metabolites into the broader regulatory network of bone homeostasis (53).

In addition to SCFAs, gut microbial metabolites such as bile acids and tryptophan derivatives also participate in bone regulation. Secondary bile acids activate TGR5 signaling to improve inflammation and bone metabolism balance, while indole−based tryptophan metabolites activate AhR signaling to protect the gut barrier and inhibit inflammatory bone loss (27, 28, 54).

Consequently, targeting SCFA production or signaling represents a promising strategy for osteoporosis treatment, aiming to harness the gut microbiota’s capacity to modulate bone metabolism via immune and barrier functions (42).

In summary, acetate, propionate, and butyrate produced by gut microbiota are vital metabolites that contribute to the maintenance of bone health by promoting bone formation, inhibiting bone resorption, and modulating immune responses and intestinal barrier integrity. Their decreased abundance in postmenopausal osteoporosis patients and positive correlation with bone mineral density highlight their potential as both biomarkers and therapeutic targets. Future research focusing on enhancing SCFA production or mimicking their effects may yield innovative interventions to mitigate bone loss and improve skeletal outcomes in estrogen-deficient populations.

### 2.5 Correlation analysis between gut microbiota composition and bone mineral density

Emerging evidence robustly indicates that the composition of gut microbiota undergoes significant alterations in postmenopausal osteoporosis (PMO) patients, which not only correlates but also exhibits a causal relationship with changes in bone mineral density (BMD) (55–57). Studies employing 16S rRNA gene sequencing and metagenomic analyses have consistently reported characteristic shifts in microbial community structure among PMO individuals compared to healthy controls. A notable hallmark is the disturbance in the Firmicutes/Bacteroidetes ratio, a key phylum-level balance that influences metabolic and inflammatory pathways relevant to bone homeostasis. For instance, PMO patients often exhibit a reduced Firmicutes/Bacteroidetes ratio, reflecting dysbiosis that may contribute to bone loss by modulating systemic inflammation and nutrient absorption (55, 58).

The associations among microbial shifts, BMD, and bone metabolic markers have been well characterized in clinical studies. Gut microbiota dysbiosis is significantly correlated with two pivotal bone turnover markers: elevated C-terminal telopeptide of type I collagen (CTX-1, a marker of bone resorption) and reduced Procollagen type 1 N-terminal propeptide (P1NP, a marker of bone formation) (47, 59, 60).Specifically, dysbiotic microbial communities promote inflammatory and metabolic disorders that enhance osteoclastic bone resorption while suppressing osteoblastic bone formation, leading to decreased BMD (47, 61, 62).As for the Firmicutes/Bacteroidetes (F/B) ratio, a lower F/B ratio (higher Bacteroidetes abundance) is generally linked to favorable metabolic health, but its correlation with BMD is complex and depends on the finer taxonomic composition within each phylum rather than the phylum-level ratio alone (55, 63, 64).

Notably, different genera within the phylum Firmicutes exert divergent regulatory effects on BMD, indicating that analysis at the genus or species level is essential rather than only at the phylum level. Within Firmicutes, Lachnospiraceae and Ruminococcaceae are positively correlated with higher BMD, as these taxa are dominant producers of butyrate and contribute to maintaining intestinal barrier function and anti-inflammatory homeostasis (55, 65–67). In contrast, some genera such as Barnesiella and Lactococcus are negatively associated with BMD and may increase the risk of osteoporosis by triggering inflammatory responses (16).

At the genus and family levels, specific taxa such as Fusobacterium, Roseburia, and Lactobacillus show significant abundance changes in PMO cohorts. Specifically, elevated Fusobacterium is significantly associated with low BMD and enhanced bone resorption, while increased Lactobacillus and Roseburia (major butyrate producers) are positively correlated with high BMD and reduced bone turnover markers (68, 69). Fusobacterium, often enriched in PMO, has been implicated in pro-inflammatory states that could exacerbate bone resorption, whereas genera like Roseburia and Lactobacillus, typically associated with short-chain fatty acid (SCFA) production and anti-inflammatory effects, tend to decrease, suggesting a loss of protective microbial functions (68, 69).

The clinical relevance of these microbial alterations is underscored by their significant correlations with BMD values and bone metabolic markers. For example, increased abundance of Lactobacillus correlates positively with higher BMD and favorable bone turnover marker profiles, indicating its potential as a biomarker and therapeutic target. Conversely, elevated Fusobacterium levels associate with lower BMD and increased bone resorption markers, highlighting its role as a risk factor. Moreover, gut microbiota diversity and richness, often reduced in PMO, have been linked with bone health parameters, although some studies report increased diversity in certain PMO populations, reflecting complex microbial dynamics influenced by host factors such as estrogen deficiency and inflammation (70).

Functional predictions based on metagenomic and metabolomic analyses reveal that gut microbiota dysbiosis in PMO is accompanied by perturbations in key metabolic pathways. Notably, abnormalities in amino acid metabolism, alkaloid biosynthesis, and lipid metabolism have been observed, which may influence bone remodeling processes through systemic metabolic effects. For instance, altered tryptophan metabolism and SCFA production affect osteoblast and osteoclast activity via immune modulation and hormonal signaling pathways (54). Additionally, gut microbiota-derived metabolites such as butyrate have been demonstrated to enhance bone mineral density by promoting osteogenesis and mitigating inflammation, further supporting the functional impact of microbial metabolic shifts on bone health (71).

Mendelian randomization studies provide genetic evidence confirming the causal link between specific gut microbiota taxa and BMD (55–57). MR analyses verified that taxa within Firmicutes (e.g., Lachnospiraceae, Clostridiales) are causally associated with higher BMD and reduced osteoporosis risk, while Barnesiella and Lactococcus are causally linked to lower BMD and increased fracture risk (56, 57). This genetic approach strengthens the concept that modulating gut microbiota composition could be a viable strategy for osteoporosis prevention and treatment (56, 57).

In summary, the gut microbiota composition in PMO patients is markedly altered, characterized by an imbalanced Firmicutes/Bacteroidetes ratio and differential abundance of key genera such as Fusobacterium, Roseburia, and Lactobacillus. These microbial shifts are not only statistically correlated but also causally linked to BMD and bone metabolic markers,These microbial shifts are significantly correlated with BMD and bone metabolic markers, suggesting their utility as biomarkers and therapeutic targets. Functional disruptions in microbial metabolic pathways further elucidate mechanisms by which gut dysbiosis influences bone metabolism. Collectively, these findings highlight the gut microbiota as a critical modulator of bone health in postmenopausal women and underscore the potential of microbiota-targeted interventions in managing osteoporosis.

### 2.6 Intestinal barrier dysfunction and endotoxin-mediated osteoporosis

Postmenopausal osteoporosis (PMO) is increasingly recognized as a multifactorial disease influenced not only by hormonal changes but also by alterations in gut barrier integrity and systemic inflammation triggered by bacterial endotoxins. After menopause, the increased intestinal permeability allows bacterial components such as lipopolysaccharide (LPS), a potent endotoxin derived from Gram-negative bacteria, to translocate into the bloodstream. This translocation initiates systemic inflammatory cascades that exacerbate bone resorption and impair bone formation. Experimental evidence from ovariectomized (OVX) mouse models, which simulate postmenopausal conditions, demonstrates that OVX induces significant disruption of the gut microbial community and compromises the intestinal barrier, resulting in elevated circulating LPS levels. The elevated LPS stimulates inflammatory cytokine production via activation of the NF-κB signaling pathway, promoting osteoclastogenesis and bone loss (72). Moreover, neuroendocrine factors such as neuropeptide Y have been shown to aggravate gut barrier dysfunction and increase serum LPS, further linking gut permeability to bone deterioration in PMO (4). This endotoxin-driven systemic inflammation represents a critical pathogenic mechanism underlying bone loss in postmenopausal women (13, 72).

Given the pivotal role of gut barrier integrity in modulating systemic inflammation and bone metabolism, therapeutic strategies aimed at restoring intestinal barrier function and reducing endotoxin translocation have emerged as promising interventions for PMO. Approaches such as dietary supplementation with prebiotics, probiotics, and bioactive phytochemicals have shown efficacy in enhancing gut barrier integrity by promoting tight junction protein expression and reducing gut permeability. For example, 2’-fucosyllactose, a well-characterized prebiotic, effectively restored gut microbial homeostasis and intestinal barrier function in aging mice, attenuating colonic inflammation and reducing systemic proinflammatory cytokines, thereby mitigating osteoporosis progression (73). Similarly, milk-derived extracellular vesicles (mEVs) have been reported to improve intestinal permeability, lower circulating endotoxin levels, and modulate gut microbiota composition favorably in OVX mice, resulting in decreased osteoclast differentiation and improved bone microarchitecture (43). These findings underscore the therapeutic potential of targeting the gut-bone axis through barrier repair and endotoxin clearance.

**Figure 1:**
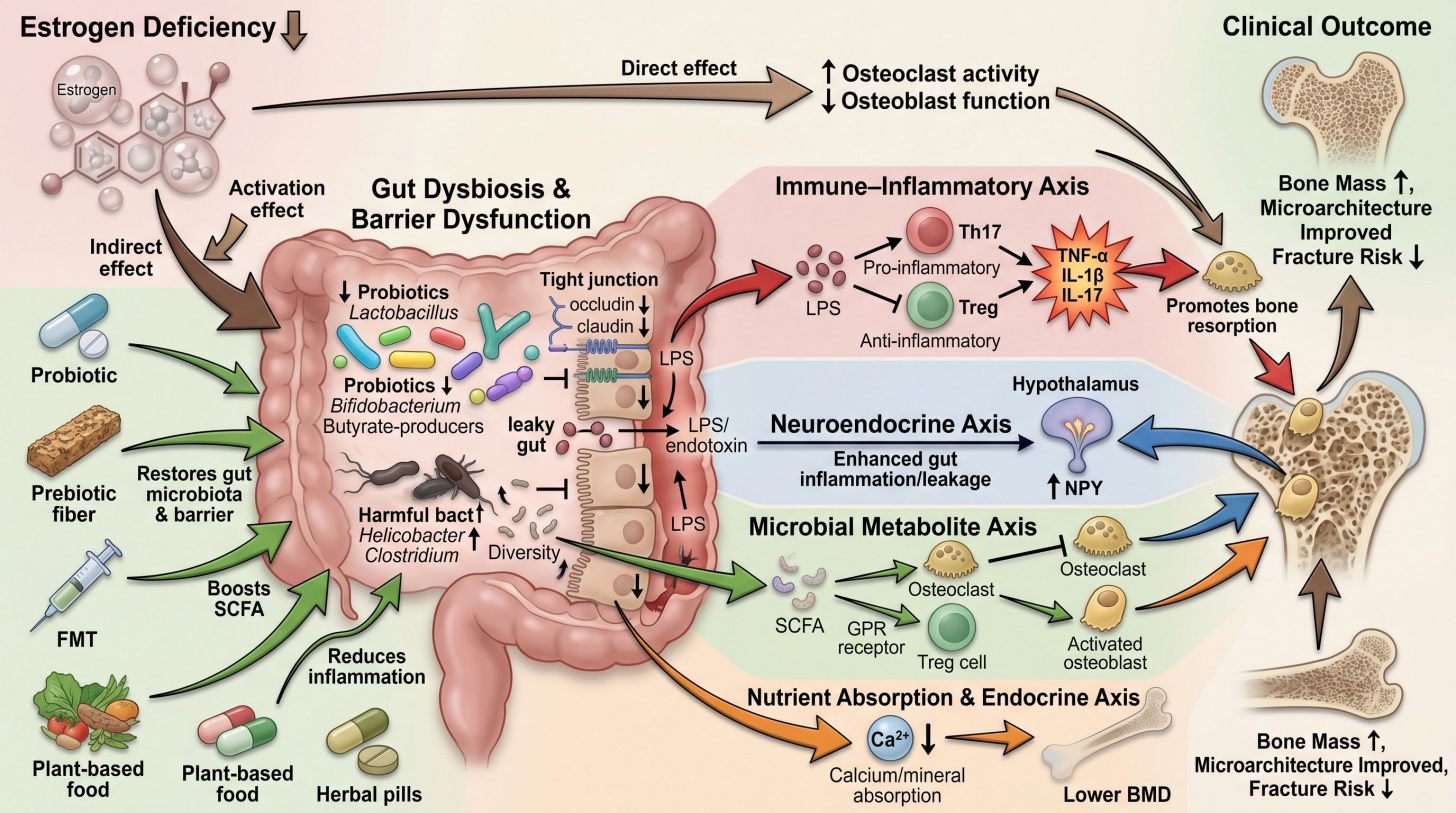
Multi dimensional regulatory network of “gut bone axis” in postmenopausal osteoporosis (PMO).

Probiotics and plant-derived phytochemicals exert bone-protective effects primarily by reinforcing intestinal barrier function and attenuating inflammation. Dietary isoquercetin, a flavonoid compound, was shown to restore gut microbial balance and enhance gut barrier integrity in OVX mice, leading to suppression of LPS-induced NF-κB activation and inflammatory cytokine release, which in turn promoted osteoblast proliferation and differentiation (72).In addition, Glycine tabacina extract demonstrated anti-inflammatory and antioxidant properties that preserved tight junction proteins and maintained intestinal epithelial integrity in models of inflammatory bowel disease, suggesting its potential utility in mitigating gut barrier dysfunction-associated systemic inflammation relevant to osteoporosis (74). The distinct roles of major SCFAs in bone metabolism are summarized in **Figure 1**. At the molecular level, these interventions reduce the translocation of endotoxins like LPS, thereby decreasing activation of proinflammatory pathways such as NF-κB and MAPK/JNK, which are implicated in both intestinal inflammation and bone resorption. Furthermore, macrophage-derived exosomal microRNAs, such as miR-223, have been identified as mediators of intestinal barrier dysfunction by targeting barrier-related proteins, highlighting novel molecular targets for intervention (75).

In summary, the disruption of intestinal barrier function after menopause facilitates the systemic dissemination of bacterial endotoxins, notably LPS, which triggers chronic low-grade inflammation and promotes bone loss characteristic of PMO. Restoration of gut barrier integrity and reduction of endotoxin burden through dietary prebiotics, probiotics, and phytochemicals represent critical therapeutic avenues. These strategies not only improve gut microbial composition and barrier function but also suppress inflammatory signaling pathways that drive osteoclast activation and inhibit osteoblast function. Continued research into the gut-bone axis and the mechanisms linking intestinal permeability to bone metabolism will be essential for developing effective interventions to prevent and treat postmenopausal osteoporosis.

## 3 Advances in clinical and experimental research on gut microbiota regulation of postmenopausal osteoporosis

### 3.1 Characteristics of gut microbiota and metabolomics research

Multiple clinical studies employing 16S rRNA gene sequencing and metabolomics techniques have revealed significant alterations in the gut microbiota diversity and metabolic profiles of postmenopausal osteoporosis (PMO) patients. These studies consistently show a reduction in gut microbial diversity in PMO, indicating dysbiosis that may contribute to disease pathogenesis. Specific bacterial taxa are altered, with decreases in beneficial genera such as Bacteroidetes and Firmicutes, and increases in potentially pathogenic groups like Proteobacteria. For example, changes in the abundance of Lachnospiraceae, Ruminococcaceae, and Faecalibacterium have been documented in various disease states, reflecting a shift in microbial community structure that may affect host bone metabolism (18, 76). Alongside microbial compositional changes, metabolomics analyses have identified abnormal levels of key metabolites in PMO patients’ gut environments. Notably, metabolites such as L-pyrroglutamic acid and bile acids show significant deviations from healthy controls, suggesting disrupted metabolic pathways linked to bone health. These metabolites are involved in crucial biological processes including amino acid metabolism and lipid digestion, which are known to influence bone remodeling and mineralization (77–79).

Metabolomic profiling further highlights significant perturbations in fatty acid metabolism and selenium compound pathways in PMO, emphasizing the role of microbial metabolites in regulating bone metabolism. Altered fatty acid metabolism can modulate inflammatory responses and osteoclast activity, thereby influencing bone resorption rates. Selenium compounds, essential for antioxidant defense, also appear dysregulated, which may exacerbate oxidative stress contributing to bone loss (26, 79, 80). These metabolic pathway disruptions underscore the complex interplay between gut microbiota-derived metabolites and host bone physiology in PMO.

Importantly, correlations have been established between gut microbiota composition and bone metabolic markers such as Procollagen type 1 N-terminal propeptide (P1NP) and C-terminal telopeptide of type I collagen (CTX-1). These biomarkers reflect bone formation and resorption activities, respectively, and their association with specific microbial taxa provides mechanistic insights into how gut microbiota may influence bone turnover. For instance, reductions in SCFA-producing bacteria correlate with decreased P1NP levels, suggesting impaired bone formation, while increases in pro-inflammatory taxa associate with elevated CTX-1, indicating enhanced bone resorption (81, 82).This evidence supports the concept of a gut-bone axis, where microbial communities and their metabolites modulate systemic bone metabolism, potentially offering novel targets for therapeutic intervention in PMO.

In summary, integrated 16S rRNA sequencing and metabolomics studies reveal that PMO is characterized by gut microbiota dysbiosis with reduced diversity and altered specific bacterial taxa, accompanied by significant changes in metabolite profiles including amino acid derivatives and bile acids. These microbial and metabolic alterations correlate with key bone turnover markers, providing a compelling basis for further mechanistic research and the development of microbiota-based strategies to prevent or treat osteoporosis in postmenopausal women.

However, most current studies focus solely on bacterial communities using 16S rRNA gene sequencing, while neglecting the fungal microbiome (mycobiome) in postmenopausal osteoporosis. The internal transcribed spacer (ITS) region is a critical target for fungal community profiling, and emerging evidence suggests that gut fungi may interact with bacteria to modulate intestinal barrier function, inflammation, and bone metabolism. In addition, both targeted and untargeted metabolomics have been widely applied to identify differential metabolites and functional pathways associated with gut dysbiosis and bone loss, providing complementary insights to microbial profiling (49). Future multi−kingdom studies combining 16S rRNA and ITS sequencing will help to reveal the complex host−microbe interactions in PMO.

### 3.2 Causal relationship between gut microbiota and osteoporosis in animal models

The ovariectomized (OVX) mouse or rat model is the most widely accepted and commonly used animal model to simulate postmenopausal estrogen deficiency and the associated bone loss. This model has become the cornerstone for verifying the causal relationship between gut microbiota dysbiosis and osteoporosis due to its stable pathological manifestations and high reproducibility.

OVX animals consistently display significant alterations in gut microbial diversity and composition compared with sham-operated controls. Typically, beneficial symbiotic bacteria, including Akkermansia muciniphila, Prevotella histicola, Lactobacillus species, and major short-chain fatty acid-producing genera, are significantly reduced, whereas potentially pathogenic and pro-inflammatory taxa such as Ruminococcus and Helicobacter are enriched (83–85). These microbial changes occur synchronously with decreased bone mineral density, deteriorated trabecular microarchitecture, and enhanced bone resorption, indicating a close connection between microbial shifts and skeletal damage.

Strong causal evidence has been provided by fecal microbiota transplantation (FMT) experiments. Transplantation of gut microbiota from osteoporotic donors, including OVX animals or postmenopausal osteoporosis patients, into germ-free or antibiotic-pretreated mice is sufficient to trigger reduced bone mass, impaired intestinal barrier function, elevated systemic inflammatory responses, and accelerated bone loss independent of estrogen signaling (84). Such findings confirm that dysbiotic microbiota alone can drive pathological bone remodeling.

The underlying mechanisms by which OVX-related gut dysbiosis promotes bone loss mainly involve integrated changes in intestinal barrier function, immune homeostasis, and microbial metabolism. Estrogen depletion impairs the expression of tight junction proteins, leading to increased intestinal permeability and translocation of lipopolysaccharide, which further activates systemic low-grade inflammation (83, 86). The inflammatory environment skews the Th17/Treg balance and stimulates the secretion of pro-osteoclastic cytokines such as TNF-α and IL-17 (84, 87). Meanwhile, the reduction in beneficial microbial metabolites, particularly short-chain fatty acids, weakens anti-inflammatory effects and support for osteoblast activity (48, 88).

Interventional studies using probiotics, prebiotics, or dietary regulation further support this causal cascade. Strategies that restore gut microbial balance in OVX models can effectively repair intestinal barrier integrity, suppress excessive inflammation, and mitigate bone loss, providing robust experimental support for microbiota-targeted therapies against postmenopausal osteoporosis (85, 88, 89).

### 3.3 Therapeutic potential of probiotics, prebiotics, and fecal microbiota transplantation

#### 3.3.1 Probiotics

Probiotics are defined as live beneficial microorganisms that confer health benefits to the host when administered in adequate amounts. Common strains investigated for bone health include species of Lactobacillus and Bifidobacterium. These strains function by regulating immune homeostasis, enhancing intestinal barrier integrity, and supporting the production of favorable microbial metabolites. Probiotics can suppress excessive inflammatory responses, inhibit osteoclastogenesis, and promote osteoblast activity, thereby attenuating bone loss in postmenopausal osteoporosis (22, 90).

#### 3.3.2 Prebiotics

Prebiotics are nondigestible dietary components that selectively stimulate the growth and activity of beneficial indigenous gut bacteria. Typical prebiotics include fructooligosaccharides (FOS), inulin, and resistant starch. By enriching beneficial taxa, prebiotics enhance gut ecosystem stability and intestinal barrier function, which indirectly modulates bone remodeling and reduces inflammation-driven bone resorption (91, 92).

#### 3.3.3 Fecal microbiota transplantation (FMT)

Fecal microbiota transplantation involves the infusion of healthy donor fecal microbiota to restore a balanced microbial ecosystem in recipients. Studies in animal models have demonstrated that FMT can reverse dysbiosis, repair intestinal barrier function, reduce systemic inflammation, and improve bone mineral density and microarchitecture (48, 93). Although still in early clinical exploration, FMT represents a promising strategy for resetting the gut ecosystem in osteoporosis. However, no human clinical trials have yet been conducted to evaluate its efficacy and safety in this context.

## 4 Overview of three intervention strategies

Probiotics offer direct microbial supplementation, prebiotics provide selective nutritional support, and FMT achieves holistic ecosystem reconstruction. Each strategy has unique advantages and limitations; combined application may yield synergistic effects for bone health protection.

### 4.1 Impact of dietary interventions on gut microbiota and bone health

Dietary patterns exert a profound influence on the composition and function of the gut microbiota, which in turn plays a pivotal role in bone metabolism and health. The intake of diets rich in prebiotics—non-digestible food components that beneficially affect the host by stimulating the growth and/or activity of advantageous bacteria—such as fruits and dairy products, has been positively correlated with bone mineral density (BMD). For instance, consumption of fiber-rich plants and fermented dairy products supports the proliferation of beneficial gut bacteria like Lactobacillus and Bifidobacterium, which enhance mineral absorption and modulate immune responses favorable to bone remodeling (60, 94).

Dietary interventions function primarily as a gut microbiota modulation strategy that shapes the overall ecosystem structure and functional capacity, rather than merely elevating SCFA levels. High-fiber diets, plant-based foods, and fermented products remodel microbial composition, enhance microbial diversity, strengthen intestinal barrier function, and regulate immune and inflammatory pathways linked to bone metabolism (42). Diets high in saturated fat, red meat, and alcohol disrupt microbial homeostasis and increase systemic inflammation, thereby accelerating bone loss. These prebiotic-rich diets support the functional stability of the gut ecosystem, which contributes to improved bone health through integrated host-microbe interactions (95–97).

T Conversely, diets high in red meat and alcohol are associated with gut microbiota dysbiosis and an increased risk of osteoporosis. Studies have demonstrated that excessive red meat and alcohol intake correlate with altered gut microbial profiles characterized by increased pro-inflammatory taxa and reduced beneficial bacteria, which may exacerbate systemic inflammation and impair bone remodeling processes. High-fat diets similarly induce gut microbial imbalances and are linked to reduced osteoblastic activity and bone formation, highlighting the detrimental impact of such dietary patterns on skeletal health. The Dietary Index for Gut Microbiota (DI-GM), a composite measure reflecting the intake of microbiota-supportive dietary components versus unfavorable ones, has been inversely associated with osteoporosis risk and fracture prevalence. Higher DI-GM scores, indicative of diets rich in fiber, fermented dairy, and plant-based foods and low in red/processed meats and refined grains, correspond to lower odds of osteoporosis and fractures, as well as modestly higher femoral neck BMD, especially among men. This evidence underscores the potential of dietary modification as a preventive strategy against osteoporosis by modulating gut microbiota composition and function. Moreover, specific functional foods such as prunes and quinoa have demonstrated bone-protective effects mediated through gut microbiota modulation, including restoration of microbial diversity and enhancement of intestinal barrier integrity, which collectively contribute to improved bone microarchitecture and metabolism in estrogen-deficient models (98–100).

An emerging and effective strategy is probiotic food matrix delivery, which uses fruits, vegetables, and fermented products as natural carriers to stabilize and deliver beneficial microorganisms to the gut. For instance, fermented dairy products combined with fruit byproducts provide a synergistic matrix that enhances probiotic survival, adhesion, and colonization efficiency. This approach improves targeted delivery of probiotics and prebiotics, thereby more effectively modulating the gut ecosystem and promoting bone health (2, 101, 102). The integration of dietary interventions targeting the gut microbiome offers promising avenues for personalized nutrition strategies aimed at mitigating postmenopausal bone loss and enhancing skeletal health.

### 4.2 Regulatory effects of phytoestrogens and traditional chinese medicine on gut microbiota

Phytoestrogens, such as soy isoflavones, and traditional Chinese medicines (TCMs) like Cordyceps species, have garnered increasing attention for their potential to modulate gut microbiota and thereby improve immune balance and bone repair, particularly in postmenopausal osteoporosis. Isoflavones, plant-derived polyphenolic compounds structurally similar to estrogen, can exert estrogenic effects by binding to estrogen receptors, which is especially relevant in estrogen-deficient states such as menopause. Studies have demonstrated that plant-based diets rich in such compounds are associated with alterations in gut microbiota composition, including increased abundance of beneficial bacteria like Akkermansia muciniphila, a mucin-degrading bacterium known for its role in maintaining gut barrier integrity and metabolic health. For example, a cross-sectional study in healthy women found that higher plant-based dietary indices correlated with increased levels of Akkermansia, suggesting that dietary phytoestrogens may influence gut microbial profiles linked to systemic metabolic and immune regulation (103). This modulation of the gut microbiota by phytoestrogens may contribute to improved bone density and strength by enhancing immune homeostasis and reducing systemic inflammation, both critical factors in osteoporosis pathophysiology.

Traditional Chinese medicines, such as Cordyceps militaris and Hericium erinaceus, contain bioactive polysaccharides and other compounds that have been shown to regulate gut microbiota composition and function. For instance, Hericium erinaceus polysaccharides improved gastrointestinal hormone secretion, enhanced intestinal barrier function, and increased beneficial bacterial populations like Lactobacillus reuteri in animal models under stress conditions, which may translate into systemic benefits including bone health (104). Similarly, Cordyceps species have demonstrated immunomodulatory effects, potentially mediated through gut microbiota alterations that favor anti-inflammatory bacterial taxa. These natural products can promote the proliferation of probiotic bacteria and suppress pathogens, thereby restoring gut microbial balance and enhancing the production of metabolites such as short-chain fattyacids (SCFAs) that have known roles in bone metabolism and immune regulation.

Emerging evidence also suggests that these natural compounds exert their effects via signaling pathways involving gut microbiota-derived metabolites. For example, plant polyphenols and polysaccharides can stimulate the secretion of gut hormones like glucagon-like peptide-1 (GLP-1), which not only regulate glucose metabolism but also modulate bone remodeling processes (105). Furthermore, the anti-inflammatory and antioxidant properties of these compounds may be partly mediated by gut microbiota modulation, leading to decreased pro-inflammatory cytokines and improved oxidative stress status, both of which are implicated in bone loss during menopause (106).

However, despite promising preclinical and clinical data, the field faces challenges that warrant further investigation. Notably, the dose-response relationships and long-term safety profiles of phytoestrogens and TCMs remain incompletely defined. Variability in bioavailability, metabolism by individual gut microbiota, and potential interactions with conventional medications complicate the translation of these findings into clinical practice (107). Moreover, the heterogeneity of gut microbiota among individuals necessitates personalized approaches to optimize therapeutic efficacy. Future research should focus on elucidating the precise mechanisms by which these natural products modulate gut microbiota and bone health, including controlled intervention trials to determine optimal dosing, duration, and safety. Additionally, advanced omics technologies could help identify key microbial taxa and metabolites mediating these effects, facilitating the development of targeted microbiota-based therapies for postmenopausal osteoporosis (100, 108, 109).

In summary, phytoestrogens such as soy isoflavones and traditional Chinese medicines like Cordyceps species hold significant potential for improving postmenopausal bone health through gut microbiota regulation. By promoting beneficial bacteria such as Akkermansia and enhancing immune and metabolic balance, these natural compounds may contribute to bone repair and the mitigation of osteoporosis. Nonetheless, comprehensive studies addressing dosage, safety, and mechanistic pathways are essential to fully harness their therapeutic benefits.

### 4.3 Genomics and single-cell technologies reveal molecular mechanisms linking gut microbiota and bone metabolism

Recent advances in genomics and single-cell sequencing technologies have substantially deepened our understanding of the molecular interplay between gut microbiota and bone metabolism, particularly in the context of postmenopausal osteoporosis (PMO). Mendelian randomization (MR) studies leveraging large-scale genome-wide association studies (GWAS) have provided compelling evidence for causal relationships between specific gut microbial taxa and bone metabolic phenotypes. Notably, the order Burkholderiales has emerged as a significant microbial group inversely associated with osteoclast activity and PMO risk. A two-sample MR analysis integrating gut microbiota GWAS data from the MiBioGen consortium and bone-related phenotypes identified Burkholderiales as causally linked to decreased osteoclastogenesis and reduced PMO susceptibility, with an odds ratio of 0.400 (P = 0.011), suggesting a protective effect (110). This finding was corroborated by parallel animal model studies demonstrating that ovariectomized mice exhibited reduced Burkholderiales abundance concomitant with increased osteoclast activity and bone loss, highlighting the microbiota-gut-bone axis as a critical regulatory pathway (83).

At the molecular level, integrative multi-omics analyses combining MR, gene expression profiling, and single-cell RNA sequencing (scRNA-seq) have identified key genes that serve as molecular bridges linking gut microbial signals to bone cell function. Two pivotal genes, FMNL2 (Formin-Like 2) and SRBD1 (S1 RNA Binding Domain 1), were pinpointed through MAGMA gene-set enrichment analysis and validated in scRNA-seq datasets of osteoclast populations. Both genes exhibited differential expression patterns correlating with osteoclast differentiation and PMO status, implicating them as critical mediators in the gut microbiota-osteoclast axis (110). FMNL2 is known to regulate actin cytoskeleton dynamics, essential for osteoclast motility and bone resorption, whereas SRBD1 is implicated in RNA binding and post-transcriptional gene regulation, potentially modulating osteoclastogenic gene networks. The identification of these genes provides mechanistic insight into how gut microbiota-derived signals may influence bone resorption processes at the cellular level.

Beyond individual gene targets, single-cell transcriptomic analyses have elucidated the cellular heterogeneity within bone marrow and gut tissues, revealing how gut microbiota dysbiosis can induce systemic inflammation and alter hematopoietic stem cell (HSC) differentiation toward myeloid-biased lineages that favor osteoclastogenesis (111). For instance, TLR9-deficient mice displayed gut microbiota alterations that triggered chronic low-grade inflammation, expansion of CD4+ T cells, and elevated pro-osteoclastic cytokines such as TNF-α, RANKL, and IL-1β, culminating in bone loss. Single-cell RNA sequencing further identified myeloid-biased hematopoiesis in bone marrow, suggesting that gut microbiota-induced immune modulation directly impacts bone remodeling dynamics (111). These findings underscore the complex immune-mediated pathways by which gut microbiota influence bone metabolism.

The integration of MR and scRNA-seq approaches also facilitates the discovery of novel molecular pathways and potential therapeutic targets. For example, gut microbiota taxa associated with ferroptosis-related proteins have been linked to osteoporosis risk, with mediation analyses highlighting proteins such as MDM4 in the gut microbiota-ferroptosis-bone axis (112). Similarly, plasma proteomics and metabolomics studies have identified circulating proteins and metabolites that mediate gut microbiota effects on bone mineral density and fracture risk, suggesting that gut microbiota modulate bone metabolism through systemic molecular intermediates (113, 114).

Collectively, these genomic and single-cell studies provide a multi-layered molecular framework elucidating how specific gut microbial taxa, such as Burkholderiales, interact with host genetic and immune factors to regulate osteoclast activity and bone homeostasis. The identification of FMNL2 and SRBD1 as key molecular bridges offers promising targets for precision interventions. These insights pave the way for developing microbiota-targeted therapies and personalized medicine approaches aimed at modulating the gut-bone axis to prevent and treat postmenopausal osteoporosis. Future research integrating longitudinal multi-omics data with functional validation will be critical to translate these molecular discoveries into clinical applications.

## 5 Conclusion

In conclusion, the intricate interplay between gut microbiota and postmenopausal osteoporosis represents a rapidly evolving frontier in bone health research. From an expert perspective, the accumulated clinical and preclinical evidence underscores the multifaceted mechanisms through which the gut microbiome influences bone metabolism—namely via neuroendocrine signaling, immune modulation, metabolic byproducts, and maintenance of intestinal barrier integrity. This complex crosstalk forms the foundation of the emerging brain-gut-bone axis concept, highlighting the gut microbiota as a pivotal regulator of skeletal homeostasis in postmenopausal women.

Balancing the diverse research perspectives, it is clear that alterations in gut microbial composition and function are closely linked to changes in bone mineral density and turnover markers. Animal models have provided mechanistic insights, while human studies have corroborated these associations, collectively validating the gut microbiota’s role as both a biomarker and a potential therapeutic target. However, heterogeneity in study designs, microbial profiling techniques, and intervention protocols necessitates cautious interpretation and calls for standardized methodologies to strengthen reproducibility and clinical translation.

Intervention strategies targeting the gut microbiome—such as probiotics, prebiotics, fecal microbiota transplantation, and dietary modulation—have demonstrated promising bone-protective effects. These approaches offer a novel adjunct or alternative to conventional osteoporosis treatments, with the potential to modulate systemic inflammation, enhance nutrient absorption, and restore microbial balance. Nonetheless, the translation of these findings into clinical practice demands rigorous, large-scale randomized controlled trials to establish efficacy, safety, optimal dosing, and long-term outcomes.

Looking forward, future research must prioritize elucidating the precise molecular mechanisms underlying microbiota-bone interactions, identifying key microbial taxa and metabolites that mediate skeletal effects, and validating these findings in diverse human populations. Integrative multi-omics approaches combined with advanced bioinformatics will be instrumental in uncovering causal relationships and therapeutic targets. Furthermore, personalized medicine frameworks should be developed to tailor microbiome-based interventions according to individual microbial profiles and risk factors.

From a policy and clinical guideline standpoint, it is imperative that decision-makers leverage the growing body of evidence to formulate rational, forward-looking recommendations that incorporate gut microbiota modulation as part of comprehensive osteoporosis management. This necessitates multidisciplinary collaboration among microbiologists, endocrinologists, nutritionists, and public health experts to ensure evidence-based, patient-centered care.

In summary, the gut microbiota stands at the forefront of a paradigm shift in understanding and managing postmenopausal osteoporosis. By harmonizing diverse research findings and advancing mechanistic and clinical investigations, the medical community can unlock the full therapeutic potential of microbiome-targeted strategies, ultimately improving bone health and quality of life for millions of postmenopausal women worldwide.
